# Effect of Melatonin Intake on Oxidative Stress Biomarkers in Male Reproductive Organs of Rats under Experimental Diabetes

**DOI:** 10.1155/2015/614579

**Published:** 2015-05-06

**Authors:** Marina G. Gobbo, Carolina F. Pereira Costa, Danilo G. Humberto Silva, Eduardo A. de Almeida, Rejane M. Góes

**Affiliations:** ^1^Department of Biology, Institute of Biosciences, Humanities and Exact Sciences, Universidade Estadual Paulista (UNESP), Cristóvão Colombo 2265, 15054-000 São José do Rio Preto, SP, Brazil; ^2^Department of Cell Biology, Institute of Biology, State University of Campinas (UNICAMP), Charles Darwin Street, Building N, 13083 863 Campinas, SP, Brazil; ^3^Department of Chemistry and Environmental Sciences, Institute of Biosciences, Humanities and Exact Sciences, Universidade Estadual Paulista (UNESP), Crisóvão Colombo 2265, 15054-000 São José do Rio Preto, SP, Brazil

## Abstract

This study investigated the antioxidant system response of male reproductive organs during early and late phases of diabetes and the influence of melatonin treatment. Melatonin was administered to five-week-old Wistar rats throughout the experiment, in drinking water (10 *μ*g/kg b.w). Diabetes was induced at 13 weeks of age by streptozotocin (4.5 mg/100 g b.w., i.p.) and animals were euthanized with 14 or 21 weeks old. Activities of catalase (CAT), glutathione-S-transferase (GST), glutathione peroxidase (GPx), and lipid peroxidation were evaluated in prostate, testis, and epididymis. The enzymes activities and lipid peroxidation were not affected in testis and epididymis after one or eight weeks of diabetes. Prostate exhibited a 3-fold increase in GPx activity at short-term diabetes and at long-term diabetes there were 2- and 3-fold increase in CAT and GST, respectively (*p* ≤ 0.01). Melatonin treatment to healthy rats caused a 47% increase in epididymal GPx activity in 14-week-old rats. In prostate, melatonin administration normalized GST activity at both ages and mitigated GPx at short-term and CAT at long-term diabetes. The testis and epididymis were less affected by diabetes than prostate. Furthermore, melatonin normalized the enzymatic disorders in prostate demonstrating its effective antioxidant role, even at low dosages.

## 1. Introduction

Diabetes mellitus (DM) affects 8.3% of the world population and approximately 5.1 million people aged between 20 and 79 years died from diabetes in 2014 [1]. The high mortality and negative impact of diabetes on life quality are due to the progressive impairment of multiple organ systems, caused mainly by hyperglycemia and oxidative stress [2]. The oxidative status in diabetes is due to impairment of mitochondrial electron transfer, the activation of polyol pathways, the catalysis of cyclooxygenase intermediate products, and enhanced nonenzymatic glycation [3–5]. In turn, advanced glycation end-products (AGE) produced by nonenzymatic glycation lead to the generation of reactive oxygen species (ROS), the activation of Bax, and expression of proapoptotic and proinflammatory genes, such as c-Jun N-terminal kinase [2, 6, 7]. Thus, as confirmed by cDNA microarray analysis, diabetes can alter the expression of multiple genes, particularly those related to cell proliferation and differentiation, oxidative stress biomarkers, DNA damage repair, and apoptosis [8].

The vast majority of patients with type 1 DM are diagnosed before the age of 30 and a modest excess of cases occurs in males [9] where its negative influence on reproductive function is relevant. Clinical evidence indicates that diabetes is associated with multiple impairment of male genital physiology, such as reduced androgen levels, erectile dysfunction, retrograde ejaculation, poor semen quality, and reduced fertility [10, 11]. Diabetes has also been associated with an increased risk of numerous cancers, but the data concerning prostate cancer are inconsistent [12, 13]. Most evidence, including a meta-analysis [14] of 19 studies published between 1971 and 2005, has indicated an inverse correlation between diabetes and prostate cancer [15, 16].

Most clinical data concerning the negative impact of diabetes on male reproductive physiology have been corroborated by experimental models of induced diabetes [17–25]. The influence of oxidative stress due to diabetes on the response of different genital organs has been previously investigated in rats [26–32]. However, information concerning how these changes occur during disease progression is scarce.

The neurohormone melatonin (N-acetyl-5-methoxytryptamine) is secreted rhythmically following a periodicity that is controlled by a circadian pacemaker located in the suprachiasmatic nucleus [33]. Melatonin (MLT) regulates several physiological functions, according to the light-dark daily cycle. It has been suggested that the rhythmicity of MLT action also controls the activity and gene expression of antioxidant enzymes [34]. MLT and its metabolites, N1-acetyl-N2-formyl-5-methoxykynuramine (AFMK), and N1-acetyl-5-methoxykynuramine (AMK) exhibit antioxidant activities that are related to the direct removal of hydroxyl radicals, nitric oxide, and peroxynitrite anions acting as free radical scavengers [35–38]. A few studies using cultured cells indicate that melatonin promoted the generation of ROS at pharmacological concentrations; however its prooxidant action in vivo remains to be elucidated [39]. Experimental evidence has shown that administration of MLT at doses of 5 mg to 150 mg/kg body weight ameliorates the oxidative status in the pancreas, liver, heart, kidneys, and testis [26, 40, 41]. Besides, the administration of low doses of MLT (25 *μ*g/mL) to rats fed with high fat-diet showed that this hormone was able to normalize the altered biochemical proinflammatory profile in these animals [42]. The consequences of MLT consumption at low doses during sexual maturation of the male genital organs and their oxidative status at adulthood are unknown. In addition, considering that MLT interferes with androgen production and affects androgen-dependent organs, which also occurs in diabetes, more information is necessary to better discriminate the putative and protective role of exogenous MLT in genital organs under diabetes and also to delineate the response of organs during disease progression. Thus, this study comparatively examined the early and advanced responses of the antioxidant system in rat male genital organs subjected to experimental diabetes and the influence of low MLT dose treatment prior to and concomitant with the disease in these systems.

## 2. Material and Methods

### 2.1. Experimental Design

**Figure 1:**
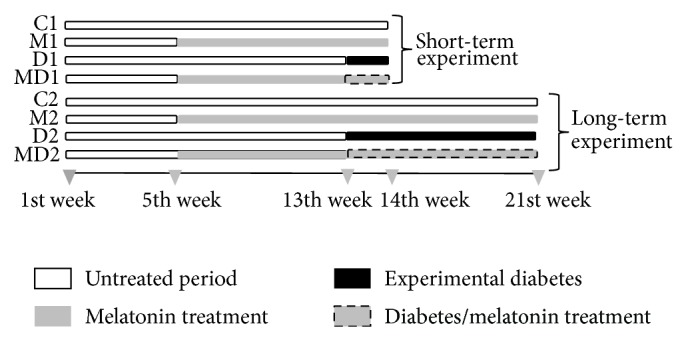
Experimental design of the study. Melatonin was offered in the drinking water (10 *μ*g/kg b.w), and diabetes was induced by streptozotocin injection (4.5 mg/100 g b.w., i.p.). C1: one-week control; M1: one-week control treated with melatonin; D1: one-week diabetic; MD1: one-week diabetic treated with melatonin; C2: two-month control; M2: two-month control treated with melatonin; D2: two-month diabetic; MD2: two-month diabetic treated with melatonin (*N* = 10 animals/group). The euthanasia was performed at 13 weeks of age for short-term experiment and at 21 weeks of age for long-term experiment.

Eighty male Wistar rats (*Rattus norvegicus*) were obtained from the breeding house of São Paulo State University (Botucatu, SP, Brazil). All experiments were performed in accordance with the Guide for the Care and Use of Laboratory Animals published by the US National Institutes of Health and acknowledged by the institutional ethical committee for animal experimentation (Protocol number 051/2011-CEEA). The animals were kept in polyethylene cages with wood shavings in a 12 : 12 light/dark cycle, at a temperature of about 22°C and with free access to food (Presence, Invivo, Paulinia, SP, Brazil) and filtered water. After an adaptation period, the rats were weighed and randomly distributed into eight groups (Figure 1, *N* = 10 per group). The short-term experiment consisted of a control (C1), a control treated with MLT (M1), one-week-diabetic rats (D1), and one-week-diabetic rats treated with MLT (MD1). The long-term experiment consisted of a control (C2), a control treated with MLT (M2), two-month diabetic rats (D2), and two-month diabetic rats treated with MLT (MD2). The administration of MLT (Sigma Chemical Co., St Louis, MO, USA) followed the procedures established by Wolden-Hanson et al. [43]. This hormone was dissolved in ethanol and stored in aliquots at −70°C. Rats in groups M1, M2, MD1, and MD2 were provided with MLT from five to 14 weeks of age, via drinking water (10 *μ*g/kg body weight in ethanol 0.001%/day). The MLT intake per day in this investigation was based on mean daily water consumption of 80 mL/day/animal and a mean body weight of 350 g and was available to the animals in plastic bottles protected from light. These conditions were standardized by the application of various consumption preference and aversion tests; therefore, this is an appropriate dosage for the induction of increased MLT levels during the night [43].

Diabetes was induced in untreated (groups D1 and D2) and MLT-treated (groups DM1 and DM2) rats aged 13 weeks. After 24 h fasting, animals were anesthetized (0.1 mL ketamine and 0.1 mL xylazine/100 g body weight) and injected intraperitoneally with 4.5 mg/100 g body weight of streptozotocin (Sigma, St. Louis, MO, USA), diluted in 0.01 M citrate buffer, pH 4.5. The control animals were injected only with citrate buffer. The blood glucose levels were evaluated two days after streptozotocin injection, in the tips of the paws using the glucose monitor Accu-chek (Roche Diagnostics, Mannheim, Germany). Only animals that showed blood glucose levels above 220 mg/dL were included in the diabetic groups. Because water consumption is higher for diabetic animals, the MLT dose was corrected for groups D1, D2, MD1, and MD2 following the diagnosis of diabetes. The C1, M1, D1, and MD1 groups were euthanized when with 14 weeks old and the C2, M2, D2, and MD2 groups were euthanized when 21 weeks old. The rats were euthanized using CO_2_ inhalation and were subsequently decapitated for blood collection.

### 2.2. Activity of Antioxidant Enzymes

The antioxidant enzyme activity of all animals was assayed in the ventral prostate, testis, and epididymis and also in blood. After dissection, these organs were weighed and homogenized in 1 : 4 volume of buffer with protease inhibitors (50 mM Tris-HCl, 1 mM EDTA, 1 mM DTT, 0.5 M sucrose, and 0.15 M KCl, 1 mM PMSF, pH 7.4) and centrifuged at 10.000 g for 20 min at 4°C. The supernatant was then recentrifuged at 50.000 g for another 60 min at 2°C and the supernatant fraction was removed and was used to measure the activity of catalase (CAT), glutathione-*S*-transferase (GST), and glutathione peroxidase (GPx).

The blood samples were collected in polyethylene tubes containing EDTA immediately after decapitating the animals. For the determination of CAT activity, the blood samples were diluted 50 times in distilled water, whereas for the determination of GPx and GST activities, blood was diluted 20 times in a hemolyzing solution (7 mM 2-mercaptoetanol; 2 mM NADP; 0.27 M EDTA).

The CAT activity was quantified at 240 nm for 1 min by the decomposition of 10 nm H_2_O_2_ [44]. The total GST activity was determined by measuring the increase in absorbance at 340 nm for 1 min 40 s by an assay containing reduced glutathione (200 mM GSH) and 1-chloro-2,4-dinitrobenzene (200 mM CNDB) as substrates, according to Keen et al. [45]. The total GPx activity was evaluated by NADPH (0.2 M) oxidation, concomitant with GSSG reduction by excess glutathione reductase, causing a decrease in absorbance at 340 nm for 1 min, according to Sies et al. [46]. All tests were performed at room temperature. The total protein content (mg/mL) in the samples was determined using bovine serum albumin as a standard, by the modified Lowry method [47]. The specific molar extinction constant (*ε*) was used to estimate the levels of enzyme activity in U/mg protein (*ε* = 0.071 for CAT, *ε* = 6.22 for GPx, and *ε* = 9.6 for GST). The equation used for enzymatic activity was [Absorbance variation × 1000/*ε*  × sample volume (*μ*L)/total protein concentration of the sample (mg/mL)].

### 2.3. Determination of Lipid Peroxidation Levels

The levels of lipid peroxidation were evaluated in the same organs and in blood, by the quantification of malondialdehyde (MDA) levels, an indicator of oxidation. For this, the presence of the colored derivative formed between MDA and 2-thiobarbituric acid (TBA) was detected via HPLC at 532 nm [48]. Quantification of MDA in the tissues was performed with 100 *μ*L of homogenized tissue in buffer (1 : 4 v/v) and 100–200 *μ*L of plasma. Three hundred *μ*L of 0.4% thiobarbituric acid solution (diluted in 0.2 M HCl) was added to the tissue and plasma samples and they were incubated for 40 min at 90°C in a dry block. The samples of TBA-MDA were extracted with 1 mL n-butanol and centrifuged at 890 g for 3 min at 3°C. TBA-MDA (20 *μ*L) samples were directly injected in HPLC and monitored at 532 nm. The mobile phase consisted of 50 nM monobasic phosphate potassium solution, pH 7.0, 20% methanol, and a pumped isocratic flow of 1.0 mL/min. The HPLC system (Shimadzu) consisted of two LC-10ADVP pumps, a SPD-M10ADVP UV-visible detector, and a SCL-10AVP controller and a LC-18 (150 × 4.6 mm, 5 *μ*m pore diameter) column was used. The MDA estimation was based on a standard calibration curve of tetramethoxypropane (TMP) previously prepared using the same procedure as that used for samples. The data were expressed as nmol/mg tissue and *μ*mol/mL plasma. The equation to calculate the amount of MDA in samples was (peak area/slope of calibration curve)/*C*,* C* = [sample volume (*μ*L) × injection volume (*μ*L))/1000 *μ*L of n-butanol].

### 2.4. Statistical Analysis

Statistical analyses were performed among groups of the same experimental period (multiple comparison tests) and between both experimental periods for the same treatment (paired difference tests) using the Statistica 9.0 software (Statsoft Inc., Tulsa, OK, USA). Data were tested for normality and homogeneity of variance assumptions according to the Shapiro-Wilk's test and Levene's test, respectively. Groups that met the assumptions (parametric data) were compared by applying a* t* test or one-way ANOVA followed by Tukey's post hoc test. Those groups that did not meet the assumptions (nonparametric data) were compared using the Mann-Whitney or Kruskal-Wallis test followed by Dunn's post hoc test. Data were expressed as mean ± standard deviation and *p* < 0.05 was considered statistically significant.

Correlation tests were conducted (Pearson's test for parametric data and Spearman's test for nonparametric data) between the levels of lipid peroxidation (MDA) and the activity of CAT, GST, and GPX, using Statistica 9.0 software (Statsoft Inc.).

## 3. Results

### 3.1. Biometric Parameters and Glycaemia

The body weights were, respectively, ~16 and ~42% lower (*p* ≤ 0.001) after one week (D1) or two months of diabetes (D2), compared to the control groups (Table 1). The MLT treatment did not affect the body weight of normal rats or the body weight loss in diabetic groups (Table 1).

The ingestion of low doses of MLT also did not influence the weight of the prostate of healthy rats (Table 1). Such treatment avoided the prostatic atrophy induced by short-term diabetes, but not by the long-term treatment (Table 1; *p* ≤ 0.05).

In both experiments, the testicular weight was not affected by diabetes independent of treatment with MLT (Table 1). The epididymal weight was higher in the M1 group compared to the diabetic groups (Table 1; *p* ≤ 0.004). Diabetes reduced the epididymal weight in both experiments (Table 1; *p* ≤ 0.04). Despite the higher epididymal atrophy confirmed in long-term diabetes, the MLT treatment prevented this atrophy (Table 1).

Animals showed blood glucose levels that were about three and six times higher after short- or long-term diabetes, respectively, than that in the control groups (*p* ≤ 0.001), regardless of MLT treatment (Table 1). Administration of MLT did not affect the glucose level homeostasis of groups M1 and M2.

### 3.2. CAT Activity

**Figure 2:**
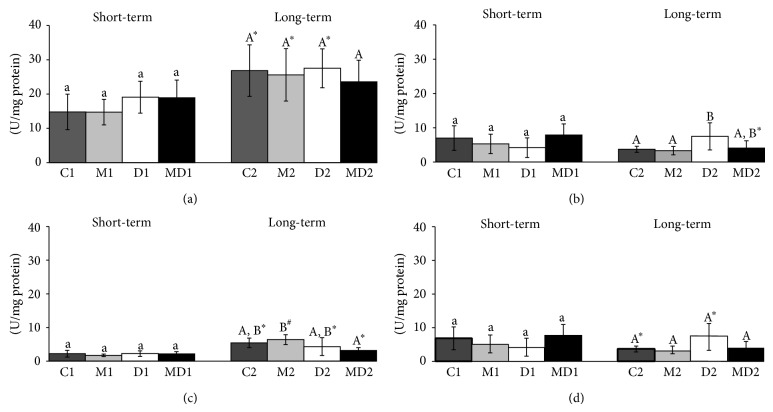
Catalase activity (U/mg protein) in blood (a), prostate (b), testis (c), and epididymis (d) of short- and long-term experiments. C1: one-week control; M1: one-week control treated with melatonin; D1: one-week diabetic; MD1: one-week diabetic treated with melatonin; C2: two-month control; M2: two-month control treated with melatonin; D2: two-month diabetic; MD2: two-month diabetic treated with melatonin (*N* = 10 animals/group). Different lowercase letters indicate statistical differences among short-term experimental groups (parametric data: (a), (b), (c), and (d)). Different uppercase letters indicate statistical differences among long-term experimental groups (parametric data: (a) and (d); nonparametric data: (b) and (c)). ∗ Indicates a statistical difference between experimental periods (parametric data). # Indicates a statistical difference between experimental periods (nonparametric data).

The CAT activity in the blood (Figure 2(a)) increased by about 40% in 21-week-old rats in comparison to the 14-week-old rats but did not change among the groups in both experiments. In the ventral prostate, the CAT activity (Figure 2(b)) was unchanged in the groups of the first experiment, but doubled after two months of diabetes compared with the control group and this increase was prevented by MLT treatment. The levels of testicular CAT were higher in the groups of long-term experiment (Figure 2(c), *p* ≤ 0.04). Melatonin had an inhibitory effect on testis CAT activity in long-term diabetes (Figure 2(c)). Similar to the testis, the activity of CAT in the epididymis of 21-week-old rats was also higher in comparison to those of 14-week-old healthy rats (*p* ≤ 0.04; Figure 2(c) and *p* ≤ 0.01; Figure 2(d), resp.).

### 3.3. GST Activity

**Figure 3:**
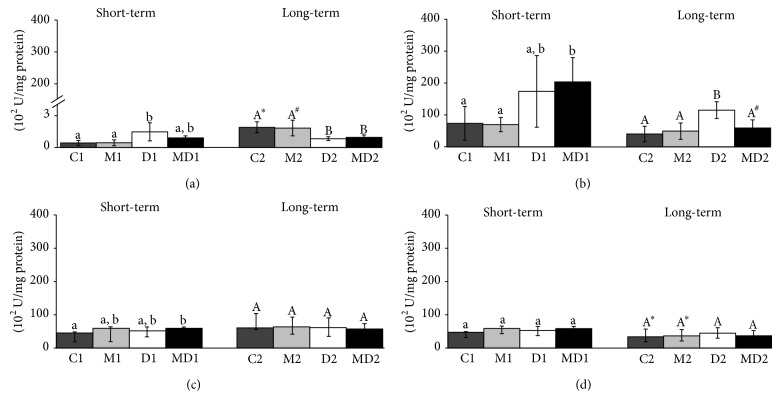
Glutathione-S-transferase activity (U/mg protein) in blood (a), prostate (b), testis (c), and epididymis (d) extracts. C1: one-week control; M1: one-week control treated with melatonin; D1: one-week diabetic; MD1: one-week diabetic treated with melatonin; C2: two-month control; M2: two-month control treated with melatonin; D2: two-month diabetic; MD2: two-month diabetic treated with melatonin (*N* = 10 animals/group). Different lowercase letters indicate statistical differences among short-term experimental groups (nonparametric data: (a), (b), (c), and (d)). Different uppercase letters indicate statistical differences among long-term experimental groups (parametric data: (c) and (d); nonparametric data: (a) and (b)). ∗ Indicates a statistical difference between experimental periods (parametric data). # Indicates a statistical difference between experimental periods (nonparametric data).

GST was the only biomarker of oxidative stress that changed in the blood after short-term diabetes (Figure 3(a)). The GST activity increased during short-term diabetes (*p* = 0.0042) and this rise was partially prevented in the MLT-treated group (Figure 3(a)); however, blood GST activity decreased (*p* = 0.0007) during long-term diabetes, regardless of the MLT treatment (Figure 3(a)). The blood GST activity increased in 21-week-old groups in comparison to younger groups (*p* ≤ 0.001). Prostatic GST activity (Figure 3(b)) also increased after the onset of diabetes (*p* = 0.0186), and MLT administration prevented this increase only in the long-term experiment. The GST activity was not affected in testis and epididymis among the animals of short and long-term experimental groups (Figure 3(d)); however the activity of this enzyme was decreased in the epididymis of older healthy rats, regardless of MLT treatment (Figure 3(d)).

### 3.4. GPx Activity

**Figure 4:**
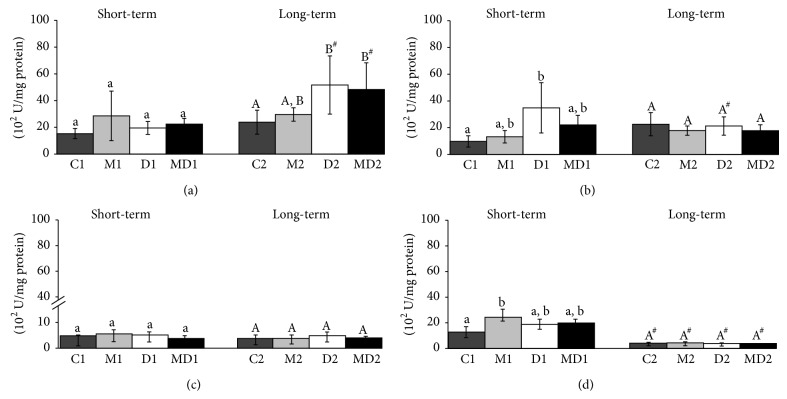
Glutathione peroxidase activity (U/mg protein) in blood (a), ventral prostate (b), testis (c), and epididymis (d) of rats. C1: one-week control; M1: one-week control treated with melatonin; D1: one-week diabetic; MD1: one-week diabetic treated with melatonin; C2: two-month control; M2: two-month control treated with melatonin; D2: two-month diabetic; MD2: two-month diabetic treated with melatonin (*N* = 10 animals/group). Different lowercase letters indicate statistical differences among short-term experimental groups (parametric data: (c); nonparametric data: (a), (b), and (d)). Different uppercase letters indicate statistical differences among long-term experimental groups (parametric data: (b), (c), and (d); nonparametric data: (a)). # Indicates a statistical difference between experimental periods (nonparametric data).

The GPx activity in blood had an inverse behavior to GST (Figure 4(a)); that is, the activity was unchanged during short-term diabetes and increased twofold after two months of diabetes compared to the control group, independent of MLT treatment. In the prostate gland, GPx activity increased ~70% one week after the onset of diabetes and MLT normalized this value in the MD1 group; however, it was unaffected in groups of the longer experiment (Figure 4(b)). The activity of this antioxidant enzyme in testis was not altered neither in experimental diabetes nor melatonin treatment (Figure 4(c)). There was an increase in GPx activity in the epididymis of healthy rats after MLT treatment in comparison to those in the control group (Figure 4(d); *p* = 0.007). Moreover, the epididymis GPx activity was very low in the two-month experiment (Figure 4(d)).

### 3.5. Lipid Peroxidation

**Figure 5:**
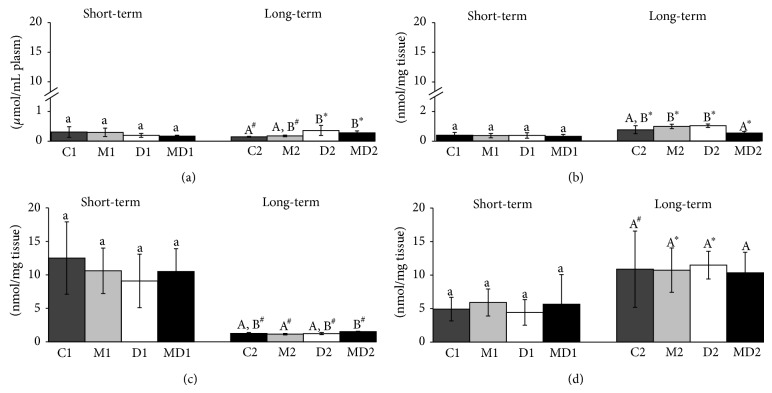
Lipid peroxidation quantified by MDA levels in plasm ((a), *μ*mol/mL plasm) and in extracts (U/mg protein) of prostate (b), testis (c), and epididymis (d). C1: one-week control; M1: one-week control treated with melatonin; D1: one-week diabetic; MD1: one-week diabetic treated with melatonin; C2: two-month control; M2: two-month control treated with melatonin; D2: two-month diabetic; MD2: two-month diabetic treated with melatonin (*N* = 10 animals/group). Different lowercase letters indicate statistical differences among short-term experimental groups (parametric data: (b), (c), and (d); nonparametric data: (a)). Different uppercase letters indicate statistical differences among long-term experimental groups (parametric data: (d); nonparametric data: (a), (b), and (c)). ∗ Indicates a statistical difference between experimental periods (parametric data). # Indicates a statistical difference between experimental periods (nonparametric data).

In blood, the MDA levels were unchanged in the groups of the short-term experiment (Figure 5(a)) and increased after two months of untreated diabetes (*p* = 0.038), regardless of MLT treatment. Diabetes did not affect lipid peroxidation in prostate (Figure 5(d)), either in the short-term or in long-term, but MLT treatment reduced by 50% the lipid peroxidation levels of long-term diabetic rats in comparison with untreated diabetic group (Figure 5(b)). Besides that, prostatic MDA levels were higher in all groups of the long-term experiment compared to the short-term groups (*p* ≤ 0.04). The testicular levels of MDA were lower (*p* ≤ 0.01) in the groups of the long-term experiment (Figure 5(c)) whereas in the epididymis, the peroxidation levels were high in these groups (Figure 5(d); *p* ≤ 0.04).

### 3.6. Correlation Tests

There was an inverse correlation between lipid peroxidation and GST activity in both short- (*r* = −0.477; *p* < 0.05) and long-term experiments (*r* = −0.669; *p* < 0.05), in blood (Figures 3(a) and 5(a)). The increase in GPx activity was directly proportional to the increase in plasma levels of MDA after two months of diabetes (*p* < 0.05 and *r* = 0.74; Figures 4(a) and 5(a)).

In the prostate, the rise in GST activity after one week of diabetes correlated inversely with the decrease in prostatic MDA levels (*r* = −0.604; *p* = 0.022, Figures 3(b) and 5(b)).

## 4. Discussion

In the present study, we delineate the comparative oxidative status in three important reproductive organs in terms of acute and chronic response to streptozotocin-induced diabetes, based on the activity of the major antioxidant enzymes and quantification of lipid peroxidation. In addition, we analyzed the effects of preadministration and prolonged use of low MLT doses on the antioxidant system of these organs and its influence on alterations caused by experimentally induced diabetes. However it is worthwhile to mention that the provision of melatonin in the drinking water, although less stressful and adequate for long-term experiments, was a limitation for this study since there is no way to know the exact amount of MLT that was ingested by each animal as well as the changes in the daily rhythm of this hormone throughout the experiment. Rasmussen et al. using a similar experimental protocol [49] verified that rats drank more than 90% of their total daily water during the dark period resulting in higher melatonin levels during the night (150.5 ± 19.2 pg/mL against 24.1 ± 8.8 pg/mL of control) but not in daytime (14.1 ± 2.6 pg/mL against 11.5 ± 0.00 pg/mL of control). As we used the same model of melatonin administration as Rasmussen et al. [49], we assume that the animals exhibited a comparable pattern of melatonin consumption and probably exhibited elevation in the levels of this hormone in dark period.

The biochemical analysis of the antioxidant system from healthy rats revealed that after 14 weeks of age, the prostate exhibited higher CAT and GST activities compared to the testis and epididymis. However, for 21-week-old rats, similar levels of antioxidant enzyme activities were detected among the three reproductive organs, with the exception of a higher GPx activity in the prostate. Therefore, biochemical analysis indicates that the levels of antioxidant enzyme activity were maintained in the prostate after a two-month interval, which was accomplished by an increase in lipid peroxidation in the gland. In contrast, the testis and epididymis showed an increase in CAT activity during aging. For the epididymis, the increase in CAT activity was accompanied by a decrease in GST and GPx activity. Taken together these findings might explain the higher susceptibility to oxidative stress of the prostate during aging, in comparison to testis and epididymis as indicated by the increase in MDA levels. These data also reinforce the organ specificity of antioxidant defense and the existence of compensatory mechanisms during aging.

The experimental protocol used here for the induction of type I diabetes is widely accepted, and most diabetic rats exhibited glucose levels above 360 mg/dL. As expected, a drastic reduction in body weight was observed, mainly in animals with chronic untreated diabetes. The body weight or blood glucose levels of normal rats were not affected by MLT treatment. Even in short-term diabetes, MLT ingestion did not normalize the glycemia or ameliorate the severe weight loss, as previously observed with doses above 10 mg/kg b.w. [28, 50, 51]. Some studies with experimental diabetes, including those with high doses of MLT, showed a normalization of blood glucose levels [40, 52]; however, this effect of MLT has been clearly reported for obese rodents in which there is an improvement in insulin sensitivity [43, 53]. Furthermore, MLT increases the metabolic activity of brown adipose tissue [54] which might favor weight loss in diabetic animals.

Streptozotocin-induced diabetes caused atrophy in the prostate and epididymis. The atrophy of these androgen-dependent organs was expected, since diabetes leads to androgen withdrawal [55, 56] and has been reported in previous studies using similar protocols for diabetes induction [28, 32, 57]. Some reports indicate a decrease in the relative testicular weight within three weeks of experimentally induced diabetes, but thereafter, testicular atrophy is no longer conspicuous [24, 57] and a similar variation was observed here, although this was not statistically significant. Treatment with MLT mitigated prostate atrophy induced by diabetes in the short-term, whereas an opposite effect was observed for the epididymis, where atrophy after short-term diabetes was not prevented, but maintenance of wet weight for long-term diabetic rats was observed. Results of our laboratory demonstrated that maintenance of epididymal weight by MLT after long-term diabetes was due to higher sperm counts in this organ (unpublished data).

The biochemical assays indicated that the prostate exhibited a more pronounced antioxidant system response to diabetes than the epididymis and testis, which were practically unresponsive. Our data demonstrate that GST participates in all phases of prostatic tissue in response to disease but early responses also involve the activation of GPx, whereas CAT activation occurs at later stages of untreated diabetes. The early activation of prostate antioxidant enzymes in response to acute diabetes in comparison to the unresponsiveness of the epididymis and testis raises several questions. This response could be due to a higher sensitivity of the prostate to streptozotocin, which is known to potentially generate free radicals [58] and might result in a direct prooxidant effect on this gland. The prostate blood barrier acts to restrict leukocyte passage into the prostatic lumen under inflammatory conditions [59]; however, the permeability of this barrier to ROS is not well known. It is also possible that the prostate antioxidant system is vulnerable to androgen regulation, as shown by in vitro studies [60], and also to the hyperglycemic status [32].

Our findings emphasize that GST is an important component in the defense against oxidative damage in the prostate. These findings agree with previous data that report a pivotal role of GST isoforms in the healthy prostate and in disease progression [61, 62]. The GSTP1 gene, which encodes the pi-class glutathione S-transferase, is a defense against oxidative damage to the genome and is expressed in high levels by epithelial cells in proliferative inflammatory Atrophy (PIA) [61–63], which are considered to be precursors of premalign and malign prostate lesions. The expression of GSTP1 is impaired in prostatic epithelial neoplasia (PIN) and neoplastic lesions, due to somatic “CpG island” DNA methylation changes [60, 63–65]. Such cells become vulnerable to oxidants and electrophiles, which result in genome damage. Furthermore, studies with transplants of tumor-cell lines demonstrated that the use of Gst-pi-siRNA suppressed the cell proliferation rate and high levels of intracellular ROS occurred in the Gst-pi knockout [63]. Experimental data from our laboratory have shown that the progression of aloxan-induced diabetes can lead to prostatic atrophy and neoplastic lesions in rats [18]. Previous studies regarding medium-term experimental diabetes showed that GST levels increased in the diabetic group and were reduced by vitamin C supplementation, which also restored rates of apoptosis in the prostate [32]. In this context, MLT treatment during diabetes prevented the increase in prostate GST, which might indicate a protective action of this neurohormone in the gland, even at low doses.

The assessment of blood-stress biomarkers was performed, to infer the systemic oxidative status. As expected, the lipid peroxidation rate indirectly demonstrated a rise in reactive oxygen species in chronic diabetes. Lipid peroxidation culminates in reduced membrane fluidity, increased nonspecific permeability, and the activation of membrane enzymes [66]. The results here and previous data [32] indicate that blood CAT activities are not altered by diabetes. In addition, they demonstrate the involvement of GST in the short-term systemic response and the suppression of its action at later stages of disease, whereas GPx exhibited the opposite behavior, with a more important role at later stages. Both GST and GPx appeared to be effective in avoiding the increase in oxidative stress due to high glucose levels, and, therefore, the levels of plasma lipid peroxidation correlated with the activity of these biomarkers although this inverse correlation was weak for MDA and GST levels.

As previously mentioned, unlike in the prostate, the response of the epididymis and testes to diabetes did not involve an increase in antioxidant enzyme activity. These results differed from data of Shrilatha and Muralidhara [28], who observed significant changes in testicular antioxidant enzymes in diabetic rats on the fifth day of exposure to the disease. These discrepancies are presumably due to small differences in streptozotocin doses, the age of the animals used, and the duration of the experiment, since this study consisted of eight weeks of experimental diabetes, compared with six weeks in that of Shrilatha and Muralidhara [28]. Surprisingly, lipid peroxidation was not affected in the testis after two months of diabetes. An increase in MDA levels was reported by Shrilatha and Muralidhara [28] in testicular mitochondria during the progression of diabetes, but no such increase for the testicular microsomal fraction was observed. This difference can be explained by the protective effect of the hematotesticular barrier and also by the existence of other oxidative stress protection mechanisms. Furthermore, our method of MDA extraction was performed using total testis homogenates, not in mitochondria and microsoma as in Shrilatha and Muralidhara [28]. Spermatozoa are very vulnerable to oxidative stress, as its polyunsaturated fatty acids in the cell membrane and nuclear and mitochondrial DNA are susceptible to oxidization [67, 68]. Furthermore, spermatozoa are very poor in free radicals scavengers [31]. The process of steroidogenesis produces ROS largely from the mitochondrial respiration chain and the catalytic reactions of the steroidogenic cytochrome P450 enzymes [69, 70]. Otherwise, within in the testis, sperm is reasonably protected from oxidative stress by the microenvironment generated by the Sertoli cells [71]. For these reasons, it is reasonable to assume that the testis provides an environment that is relatively well protected against oxidative stress, as observed here.

Sperm maturation in the epididymis necessitates a certain level of oxidation, because ROS appear to be key modulators of the early signal transduction mechanisms that lead to capacitation [72]. Thus, a fine equilibrium between beneficial oxidation and detrimental oxidative damage has to be maintained in the epididymal environment [73]. Previous reports indicated that CAT does not appear to be a major participant in the control of oxidative stress in this organ [74], whereas GPx have been implicated in this process [75] and its expression is regulated by androgens [76]. Except for the increase of GPx activity in epididymis of healthy rats treated with MLT for 9 weeks, our data indicated no marked variations in activity of antioxidant enzymes and lipid peroxidation levels under diabetes and reinforce the importance of fine adjustment of epididymal antioxidant system [72, 73] and its resilience to experimental diabetes.

Several studies have demonstrated the protective action of MLT against ROS during aging in various organs [77–79]; however, there is no information concerning organs of the male genital system. These biochemical assays indicated that treatment with low MLT doses did not affect changes in the antioxidant system during aging, except for a discrete reduction in CAT activity in the epididymis.

The present research demonstrates that hyperglycemia can adversely affect the antioxidant defense of blood and tissue extracts, particularly of the prostate. The antioxidant system of the testis and epididymis is less vulnerable to diabetes effects and is probably related to intrinsic characteristics of histophysiology and to the expression pattern of antioxidant enzymes. The MLT treatment mitigated the rise in blood GST activity during the early phase of diabetes. This treatment was more effective for the prostate, mainly in the longer experiment, as demonstrated by normalization of CAT and GST activities and MDA levels. Glutathione-*S*-transferase proved to be a good marker of compensatory antioxidant defense in the ventral prostate, corroborating our previous data on medium-term diabetes. Melatonin normalized the activities of antioxidant enzymes in the prostate, even at low doses, which demonstrates its effective antioxidant role in this organ.

## Figures and Tables

**Table 1:** The mean and standard deviation of body, prostate, testis, and epididymis wet weight and blood glucose levels of short- and long-term experimental groups.

	C1	M1	D1	MD1
Body weight (g)	383.27 ± 39.92^a^	390.85 ± 66.88^a^	320.94 ± 46.91^b^	293.85 ± 28.67^b^
Prostate weight (mg)	371.57 ± 36.04^a^	346.1 ± 42.28^a,b^	288.53 ± 68.19^b^	328.68 ± 82.24^a,b^
Testis weight (g)	1.694 ± 0.10^a,b^	1.795 ± 0.22^a^	1.629 ± 0.19^a,b^	1.570 ± 0.09^b^
Epididymis (mg)	540.86 ± 0.06^a,b,c^	590.39 ± 0.09^a^	494.22 ± 0.11^b,c^	496.58 ± 0.06^c^
Blood glucose levels (mg/dL)	111.21 ± 33.10^a^	102.85 ± 16.63^a^	359.66 ± 79.48^b^	404.18 ± 32.36^b^

	C2	M2	D2	MD2

Body weight (g)	451.71 ± 16.14^A#^	450.57 ± 40.72^A,C∗^	259.66 ± 14.22^B#^	295.2 ± 59.33^B,C^
Prostate weight (mg)	613.4 ± 89.0^A∗^	543.33 ± 67.58^A∗^	219.6 ± 50.53^B^	231.5 ± 34.88^B∗^
Testis weight (g)	1.812 ± 0.09^A∗^	1.701 ± 0.21^A^	1.603 ± 0.24^A^	1.504 ± 0.30^A^
Epididymis (mg)	728.64 ± 0.05^A∗^	612.35 ± 0.15^A,B^	466.04 ± 0.05^B^	733.75 ± 0.17^A,B∗^
Blood glucose levels (mg/dL)	87.85 ± 11.12^A#^	89 ± 9.05^A^	491.16 ± 56.55^B∗^	483.4 ± 84.2^B∗^

C1: one-week control; M1: one-week control treated with melatonin; D1: one-week diabetic; MD1: one-week diabetic treated with melatonin; C2: two-month control; M2: two-month control treated with melatonin; D2: two-month diabetic; MD2: two-month diabetic treated with melatonin (*N* = 10 animals/group). ^∗^Different lowercase letters indicate statistical differences among short-term experimental groups (parametric data: prostate and epididymis weight; nonparametric data: body and testis weight and blood glucose levels). Different uppercase letters indicate statistical differences among long-term experimental groups (parametric data: prostate weight; nonparametric data: body, testis and epididymis weight, and blood glucose levels). ^∗^Indicates a statistical difference between experimental periods (parametric data). ^#^Indicates a statistical difference between experimental periods (nonparametric data).
